# Preparation and characterization of metal-substituted carotenoid cleavage oxygenases

**DOI:** 10.1007/s00775-018-1586-0

**Published:** 2018-06-26

**Authors:** Xuewu Sui, Erik R. Farquhar, Hannah E. Hill, Johannes von Lintig, Wuxian Shi, Philip D. Kiser

**Affiliations:** 1 Department of Pharmacology, School of Medicine, Case Western Reserve University, 10900 Euclid Ave., Cleveland, OH 44106 USA; 2 National Synchrotron Light Source-II, Brookhaven National Laboratory, Upton, NY 11973 USA; 3 Center for Proteomics and Bioinformatics, Center for Synchrotron Biosciences, School of Medicine, Case Western Reserve University, 10900 Euclid Ave., Cleveland, OH 44106-4988 USA; 4 Cleveland Center for Membrane and Structural Biology, Case Western Reserve University, 1819 E 101st Street, Cleveland, OH 44106 USA; 5 Research Service, Louis Stokes Cleveland VA Medical Center, 10701 East Boulevard, Cleveland, OH 44106 USA; 6 Present Address: Department of Genetics and Complex Diseases, Harvard T.H. Chan School of Public Health, Boston, MA 02115 USA

**Keywords:** Non-heme iron enzyme, Cobalt substitution, X-ray absorption spectroscopy, Crystal structure

## Abstract

Carotenoid cleavage oxygenases (CCO) are non-heme iron enzymes that catalyze oxidative cleavage of alkene bonds in carotenoid and stilbenoid substrates. Previously, we showed that the iron cofactor of CAO1, a resveratrol-cleaving member of this family, can be substituted with cobalt to yield a catalytically inert enzyme useful for trapping active site-bound stilbenoid substrates for structural characterization. Metal substitution may provide a general method for identifying the natural substrates for CCOs in addition to facilitating structural and biophysical characterization of CCO-carotenoid complexes under normal aerobic conditions. Here, we demonstrate the general applicability of cobalt substitution in a prototypical carotenoid cleaving CCO, apocarotenoid oxygenase (ACO) from *Synechocystis*. Among the non-native divalent metals investigated, cobalt was uniquely able to stably occupy the ACO metal binding site and inhibit catalysis. Analysis by X-ray crystallography and X-ray absorption spectroscopy demonstrate that the Co(II) forms of both ACO and CAO1 exhibit a close structural correspondence to the native Fe(II) enzyme forms. Hence, cobalt substitution is an effective strategy for generating catalytically inert but structurally intact forms of CCOs.

**Electronic supplementary material:**

The online version of this article (10.1007/s00775-018-1586-0) contains supplementary material, which is available to authorized users.

## Introduction

Carotenoid cleavage oxygenases are an ancient family of enzymes that catalyze the oxidative scission of alkene substrates [1]. These include carotenoids and apocarotenoids but also metabolically unrelated compounds such as stilbenoids and other phenylpropanoids [2]. Mammalian genomes encode two such enzymes with documented oxidative cleavage activity [3]. The first to be identified is an enzyme known as β-carotene oxygenase 1 (BCO1), which cleaves β-carotene symmetrically to form retinal. The second, BCO2, cleaves at the 9–10 (9′–10′) position of the polyene chain in a number of related non-cyclic and bi-cyclic carotenoids such as lutein and zeaxanthin, respectively. Substrate specificity is similarly well established in the CCOs of higher plants which comprise two broad subclasses catalyzing the cleavage of all-*trans* or 9-*cis*-carotenoids/apocarotenoids to form chromophores, volatiles and signaling molecules including abscisic acid and strigolactone precursors [4–7]. Microbial CCOs also can cleave various carotenoid and apocarotenoid substrates although, in many cases, the physiological substrates remain poorly defined [8]. Similarly, the biological roles and biologically relevant substrate specificity of stilbenoid-cleaving CCOs, which are confined to eubacteria and fungi, remain relatively poorly understood despite their potential importance in lignin degradation and pathogen defense mechanisms against phytoalexins such as resveratrol [9, 10].

The oxidative cleavage of alkene bonds by CCOs is accomplished through the use of a mononuclear non-heme iron center [11]. The iron cofactor presumably mediates reductive activation of dioxygen (O_2_) to overcome the kinetic barrier associated with spin inversion of the O_2_ triplet ground state during its reaction with singlet organic molecules [12]. The CCO iron coordination motif is unique among non-heme iron proteins characterized to date. It consists of an absolutely conserved set of four His residues that are arranged in a pseudo-octahedral geometry, three of which are engaged in hydrogen bonds with outer sphere Glu residues that are also highly conserved within the family [13]. The iron is thus coordinatively unsaturated by protein ligands leaving two sites localized in *cis* potentially available for ligand binding. One of these sites cannot readily accommodate ligands in most of the CCOs characterized to date due to the close proximity of a Thr or Val residue methyl group. The other location, which is in direct contact with the substrate binding site, binds an aquo or hydroxo ligand in the resting state of these enzymes and is presumably also the binding site for O_2_ during catalysis. The mode of O_2_ binding to the iron cofactor in CCOs has been studied computationally [14], but despite modeled O_2_ molecules in CCO crystal structures ([15, 16], but see [17]) the CCO iron-oxy complex remains poorly characterized from an experimental standpoint.

Likewise, until recently, limited information was available on the mode of substrate binding to the CCO active site and the proximity of the scissile double bond to the iron cofactor and its O_2_ binding site. Carotenoids are hydrophobic compounds and thus inherently poorly water soluble. The limited concentrations of the compounds achievable in aqueous solution thwarted attempts to form ES complexes in sufficient yield for crystallographic studies [11, 18]. These barriers have been overcome by two advances. The first is the use of substrate/transition state analogs with improved water solubility as demonstrated for the RPE65 subgroup of CCOs [19] and later through the use of stilbene-cleaving CCOs, whose substrates are naturally more water soluble compared to carotenoids, as structural targets. In the latter case, co-crystallization of a native iron enzyme in this subfamily (*Novosphingobium aromaticivorans* carotenoid oxygenase 1, NOV1) with resveratrol led to tenuously supported active site electron density for this compound, likely due to a mixture of un-cleaved substrate, reaction intermediates and cleavage products all being present within the crystal [16, 17]. The second advance was the generation of a cobalt-substituted stilbene-cleaving CCO from *Neurospora crassa* (CAO1) that was shown to be catalytically inert and allowed the crystallographic observation of well-resolved enzyme–substrate complexes [20].

Metal substitution has previously been used as a strategy to study the structure and function of mononuclear non-heme iron oxygenases [21]. Manganese and cobalt have been used most frequently in such investigations owing to similarities in their preferred coordination geometries, ionic radii and stable ionization states. Mn(II) and Co(II) both have elevated reduction potentials relative to Fe(II), which disfavors the formation of oxidized metal species that are critically involved in O_2_ activation [22]. Hence, Mn(II)- and Co(II)-substituted versions of mononuclear non-heme iron dioxygenase are typically catalytically inactive. Important exceptions to this generalization are some extradiol dioxygenases and cupin-family dioxygenases which are active in Mn(II) and Co(II) forms [23–25].

The success of metal substitution strategies in the study of non-heme iron dioxygenase structure and reaction chemistry suggests that this approach might also be viable for the study of CCO-carotenoid interactions which to date have resisted atomic level structural characterization. In this study, we examine the susceptibility of a prototypical carotenoid-cleaving CCO, *Synechocystis* apocarotenoid oxygenase (ACO), to metal-substitution and ascertain the effects of such replacement on the catalytic properties and structure of CCOs. The results validate the use of Co-substituted CCOs to infer biological properties and chemical reactivity.

## Materials and methods

### Reagents

Except as noted below, chemical reagents were purchased from Sigma-Aldrich (St. Louis, MO) or USB Biochemicals (Cleveland, OH) in the highest purity form available. Water from a Milli-Q purification system (resistivity 18.2 mΩ cm) (ED Millipore, Billerica, MA) was used to prepare all reagents and buffered solutions.

### Expression and purification of metal-substituted ACO

All glassware and containers were extensively rinsed with Milli-Q water to minimize potential metal contamination. Expression of native, iron-containing ACO from lysogeny broth (LB) media was performed according to a published method [18]. For the production of non-native metal ACO, a pET3a plasmid containing the coding sequence of ACO (Diox1, GenBank BAA18428.1) from *Synechocystis* sp. PCC6803 was transformed into the T7 express BL21 *Escherichia coli* strain (New England Biolabs, Ipswich, MA). Two milliliter of LB media containing 100 μg/mL ampicillin were inoculated with fresh bacterial colonies from an LB agar plate containing 100 μg/mL ampicillin and grown for ~ 5 h at 37 °C with shaking at 235 rpm. Then the cells were collected by centrifugation at 3220*g* for 15 min at 37 °C. The cell pellets were resuspended in 1 mL of M9 minimal media (1X M9 salts, 2 mM MgSO_4_, 0.4% w/v glucose, 0.8% v/v glycerol and 0.1 mM CaCl_2_) and transferred into 500 mL of the same media containing 100 μg/mL ampicillin. Cells were grown at 37 °C with 235 rpm shaking to an OD_600 nm_ of ~ 0.5 at which time the temperature was lowered to 28 °C and an additional 100 μg/mL of ampicillin were added to the culture. Protein expression was induced by addition of isopropyl β-d-1-thiogalactopyranoside to a final concentration of 100 μM. At the time of induction, 15 mg of different metal salts (CoCl_2_·6H_2_O, MnCl_2_·4H_2_O, CuCl_2_·2H_2_O, (NH_4_)_2_Fe(SO_4_)_2_·6H_2_O) were added into the 500 mL culture for intended production of metal-substituted ACOs. One culture was grown without any metal supplementation for production of metal-free ACO (apo-ACO). Cells were harvested by centrifugation after overnight growth, resuspended in 20 mM HEPES–HCl, pH 7 buffer and stored at − 80 °C. ACO purification was carried out as previously described [18]. Co-CAO1 was expressed in minimal media containing CoCl_2_ and purified as previously described [20]. Purified protein samples were either flash-frozen and stored in liquid nitrogen or placed on ice for immediate use.

### ACO activity assays

The enzymatic activity of metal-free, native and putative metal-substituted forms of ACO was measured by HPLC as previously described with minor changes [18]. Specifically, 2 μg of purified ACO were added to a 200 μL reaction buffer consisting of 20 mM HEPES–NaOH, pH 7 and 0.05% (w/v) Triton X-100 (Anatrace, Maumee, OH). Reactions were initiated by addition of an ethanolic solution of all-*trans*-8′-apocarotenal to a final concentration of 100 μM. Reaction mixtures were placed in a shaker-incubator operating at 28 °C with 500 rpm shaking for 1 min and then quenched by addition of 300 μL of methanol. The reaction products, all-*trans*-retinal and 8′-hydroxy-15′-apocarotenal, as well as the remaining substrate were extracted with 500 μL of hexane and analyzed directly by high-performance liquid chromatography as previously described [18]. The amount of generated all-*trans*-retinal was quantified by comparison against a standard curve that was generated with known amounts of all-*trans*-retinal (Toronto Research Chemicals, Toronto, Canada, > 95% purity).

ACO activity was also measured spectrophotometrically using a Lambda Bio spectrometer (Perkin Elmer, Waltham, MA) or a Flexstation 3 plate reader (Molecular Devices, San Jose, CA) as previously described with minor changes [26, 27]. Six microgram of purified ACO was added to 100 μL of reaction buffer with or without test compounds. Following a 10 min incubation period at 28 °C, all-*trans*-8′-apocarotenal was added to the enzyme at a final concentration of 25 μM in a reaction volume of 200 μL. Assays were also performed with protein omitted to determine the rate of non-enzymatic loss of apocarotenoid substrate.

### Analysis of metal content

The transition metal content of each ACO sample was measured by inductively coupled plasma optical emission spectroscopy (ICP-OES) analyses at the University of Minnesota Soil Research Analytical Laboratory. Samples for ICP-OES analysis were prepared by digesting each purified, putatively metal-substituted ACO sample in 1% v/v HNO_3_. The proteins precipitated immediately upon addition of HNO_3_ and the samples were incubated for 3 h at room temperature with gentle rocking. Protein precipitates were then removed by filtration through a 0.22 μm membrane (Millipore) and the aqueous solutions that contained dissociated metals were analyzed by ICP-OES. The buffer solution was treated identically to determine the background metal content. A summary of the metal analyses for each sample is shown in Table 1. To accurately quantify the protein concentration in the samples and thus the protein–metal stoichiometry, amino acid quantification was performed on purified ACO (Texas A&M University, Protein Chemistry Laboratory). The molar extinction coefficient of ACO was determined to be 75,249 M^−1^ cm^−1^ at 280 nm [27].

**Table 1:** ICP-OES of ACO preparations

Expression media	Protein concentration (mg/mL)	Metal concentration (ppm)				Target metal/ACO
		Fe	Co	Cu	Mn	
LB	1.097	1.522	< 0.01	0.043	< 0.01	1.349
M9 + Fe	1.847	1.338	< 0.01	0.021	< 0.01	0.704
M9 + Co	1.234	< 0.01	1.075	0.037	< 0.01	0.802
M9 + Cu	0.382	0.012	< 0.01	0.284	< 0.01	0.635
M9 + Mn	1.508	0.378	< 0.01	0.018	0.018	0.012
M9	0.722	0.034	< 0.01	0.033	< 0.01	–

### Optical spectroscopy

Optical spectra of ACO samples were recorded at ambient room temperature with a Lambda Bio spectrometer (Perkin Elmer) using micro UV-transparent cuvettes (Perkin Elmer). Samples of ACO generated in the absence of added iron or cobalt were used to subtract background absorbance in the 500–600 nm wavelength range of the Co-ACO sample to isolate the weak Co-associated *d*–*d* absorption bands. This was accomplished by scaling the apo-ACO 417 nm absorption peak to match that of the 429 nm Co-ACO absorption peak and then subtracting the resulting spectra. Spectra were scaled and plotted using Excel (Microsoft, Redmond, WA) and Sigmaplot (Systat, San Jose, CA).

### Protein crystallization, structural determination and analysis

Prior to initiation of crystallization trials, purified Co-ACO samples were loaded onto a 25 mL Superdex 200 gel filtration column (GE Healthcare, Little Chalfont, UK) equilibrated with buffer containing 20 mM HEPES–NaOH, pH 7.0, and 0.02% (w/v) Triton X-100. The protein eluted in a single, symmetrical peak at ~ 13 mL. Protein-containing fractions within this elution peak were collected and concentrated to 8–10 mg/mL. Crystallization was performed by the hanging-drop, vapor-diffusion method by mixing 1 μL of purified ACO with 1 μL of reservoir buffer containing 0.1 mM Bis–tris propane–HCl, pH 6.0, 18–22% (w/v) sodium polyacrylate 2100, and 0.2 M NaCl. Crystallization trays were prepared at room temperature and then placed in an 8 °C incubator. Rod-shaped crystals appeared within 1–2 weeks and grew to final dimensions of ~ 100 × 100 × 500 μm. Mature crystals were cryoprotected by soaking in the reservoir solution supplemented with 10% glycerol and flash cooled in liquid nitrogen before X-ray exposure. Co-CAO1 was crystallized and cryoprotected as previously described [20]. Diffraction data were collected on beamline X29 at the National Synchrotron Light Source (Co-ACO crystals) or NECAT beamline 24-ID-E of the Advanced Photon Source (Co-CAO1 crystals). For Co-ACO crystals, data were collected at the wavelength of peak flux, as well as at wavelengths above and below the cobalt K absorption edges to allow assessment of Co active site occupancy. Data sets were processed with XDS [28]. Structures were determined by direct refinement using previously determined isomorphous structural models (PDB accession codes: 4OU8 for Co-ACO and 5U8Y for Co-CAO1). Initial models were then subjected to multiple rounds of manual model rebuilding and updating in Coot [29] followed by restrained refinement in Refmac [30]. Refmac input files were prepared with the CCP4 interface [31]. The stereochemical quality of the models was assessed with the Molprobity [32] and wwPDB validation server [33]. Anomalous log-likelihood gradient maps were computed using Phaser [34]. A summary of the X-ray data and refinement statistics is shown in Table 2. All structural figures were prepared with PyMOL (Schrödinger, New York, NY).

**Table 2:** X-ray diffraction data collection and structure refinement statistics

Crystal	Co-ACO	Co-ACO post edge^b^	Co-ACO pre edge^b^	Co-CAO1
*Data collection and processing* ^a^				
X-ray source	NSLS X29	NSLS X29	NSLS X29	NECAT 24IDE
Wavelength (Å)	1.07500	1.60210	1.61460	0.97946
Space group	*P*2_1_2_1_2_1_			*P*3_2_21
Unit cell parameters (Å)	118.07, 124.53, 202.61	118.75, 124.82, 202.66	118.78, 124.93, 202.84	100.69, 448.38
Resolution (Å)^c^	48.4–2.21 (2.34–2.21)	48.51–2.82 (2.99–2.82)	48.54–2.81 (2.98–2.81)	50–2.2 (2.33–2.2)
Unique reflections	145,134 (23,929)	138,304 (21,680)	139,028 (21,919)	133,339 (20,826)
Multiplicity	6.3 (6.0)	3.8 (3.8)	3.8 (3.7)	3.4 (3.3)
Completeness (%)	97 (88)	98.3 (95.6)	98.3 (95.7)	98 (96.1)
$\langle I/\sigma I\rangle$	15.3 (1.2)	10.5 (1.22)	8.7 (0.8)	10.4 (1.1)
*R*_merge_*I* (%)	10.2 (181)	13 (107.4)	17.4 (157.6)	7.9 (108.9)
CC_1/2_ (%)	99.9 (79.2)	99.2 (48.9)	98.6 (33.6)	99.8 (41.9)
Wilson *B* factor (Å^2^)	52.6	52.6	54.6	52.9
*Refinement* ^d^				
Resolution (Å)	48.4–2.21			49.1–2.2
No reflections	135,096			127,272
*R*_work_/*R*_free_ (%)	20.8/22.9			17.9/20.5
Total atoms	15,788			16,883
Protein atoms	15,100			15924
Active site metals	4			4
Water	681			955
〈*B*-factor$\rangle$ (Å^2^)	59.9			53.2
Protein	60.2			53.3
Active site metals	37.5			44.2
Water	54.0			50.4
RMS deviations				
Bond lengths (Å)	0.013			0.009
Bond angles (º)	1.55			1.36
Ramachandran plot (% favored/outliers)^e^	98.2/0			98/0
Molprobity score (%)	100			100
PDB accession code	6BIG			6B86

^a^Data were processed with XDS [28]

^b^Friedel pairs unmerged

^c^Numbers in parentheses are for the highest resolution shell of data

^d^Refinement was carried out using REFMAC [30]

^e^Ramachandran analysis was carried out using Molprobity [32]

### Sample preparation for X-ray absorption spectroscopy

Purified Co-ACO (> 95% purity by SDS-PAGE) was concentrated to a cobalt content of ~ 1.28 mM in 20 mM HEPES–NaOH, pH 7.0 containing 20% glycerol. Purified Co-CAO1 (> 95% purity by SDS-PAGE) was concentrated to a cobalt content of 0.75 mM in 16 mM HEPES pH 7, 160 mM NaCl and 20% glycerol. Samples were flash cooled in liquid nitrogen and stored on dry ice before use. Samples were loaded into copper sample cells (NSLS) or Delrin cuvettes (SSRL) wrapped in Kapton tape and flash frozen in liquid nitrogen immediately prior to X-ray absorption spectroscopy (XAS) measurements.

### XAS data collection

XAS data were obtained at the National Synchrotron Light Source (NSLS) on beamline X3B (ACO) and the Stanford Synchrotron Radiation Lightsource on beamline 9-3 (CAO1). At NSLS, the storage ring operated at 2.8 GeV and 180–300 mA. A Si(111) double crystal monochromator with sagittal focusing of the second crystal provided energy selection and horizontal focusing, with a downstream Ni-coated cylindrically bent mirror rejecting higher harmonics and providing vertically focusing. The Ni coating restricted the maximal photoelectron wave vector value (*k*_max_) for cobalt EXAFS to ~ 12.0 Å^−1^. At SSRL, the storage ring operated at 3.0 GeV and 500 mA in top-off mode. A Si(220) double crystal monochromator provided energy selection, with an upstream Rh-coating collimating mirror rejecting higher harmonics prior to the monochromator and a downstream Rh-coated toroidal mirror focusing the monochromatic radiation at the sample. Temperature control was provided by cryostats of either the He Displex type (NSLS, 15–20 K sample temperature) or Oxford liquid He design (SSRL, 10 K sample temperature). XAS data were acquired in fluorescence mode using Canberra solid state germanium detectors (NSLS: 31-element discrete; SSRL: 100 pixel monolithic) with XIA digital data acquisition electronics. Data were typically acquired in 10 eV steps in the pre-edge region (1 s acquisition time), 0.3 eV steps along the edge (2 s acquisition time), and 0.05*k* steps in the EXAFS up to 14–15*k* (acquisition time increasing from 2–9 s in a *k*^2^ weighted fashion). Samples were monitored for evidence of radiation damage as indicated by red-shifts in either the pre-edge or edge energies, and new spots were exposed as needed. A cobalt metal foil placed between ion chambers downstream of the sample was used for energy calibration, with the first inflection point of the edge set to 7709.0 eV.

### XAS data analyses

XAS data were processed and averaged using Athena [35] for NSLS X3B data, whereas EXAFSPAK [36] was used for SSRL 9-3 data. Pre-edge peak fitting analysis was carried out with Fityk using a previously reported protocol [20] over the energy range of 7702–7718 eV. Extended X-ray absorption fine structure (EXAFS) analysis was performed with Artemis [35]. Theoretical phase and amplitude parameters were calculated for a cobalt-substituted model of the Fe-ACO active site using FEFF6L [37]. From this, relevant paths were incorporated into the fitting model and evaluated for the significance of their contributions. For a given shell in all simulations, the coordination number *n* was fixed, while *r* and *σ*^2^ were allowed to float. The amplitude reduction factor *S*_0_^2^ was fixed at 0.9, while the edge shift parameter Δ*E*_0_ was allowed to float at a single common value for all shells. Histidine multiple scattering was evaluated using a method where the imidazole moiety is represented by four sets of grouped paths representing the dominant scattering contributions [38]. The fit was evaluated in *k*^3^-weighted *R*-space, and fit quality was judged by the reported *R*-factor and reduced *χ*^2^.

## Results

### ACO is robustly expressed in minimal media containing non-native metals

**Fig. 1:**
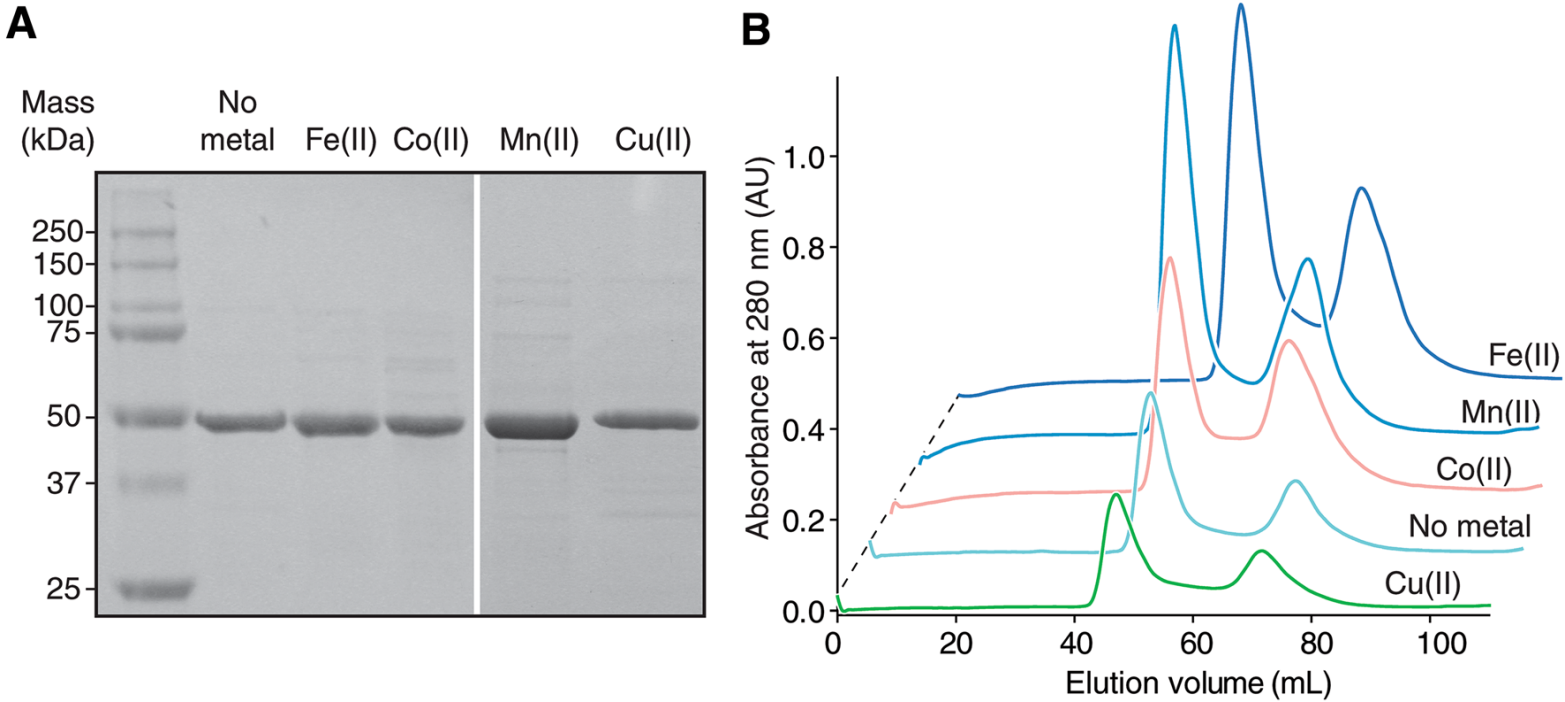
Expression and purification of ACO cultured in minimal media with or without added metals. **a** ACO samples obtained after ammonium sulfate fractionation. Precipitated proteins were re-dissolved in 20 mM HEPES–NaOH, pH 7.0, separated by SDS-PAGE and visualized by Coomassie Blue staining. The primary band of each sample migrated at ~ 50 kDa, slightly lower than the actual 54 kDa ACO molecular mass as described previously [18]. **b** Chromatograms (*A*_280 nm_) recorded during purification of the ACO samples shown in **a** by gel filtration chromatography. The monomeric ACO used for subsequent studies eluted at approximately 75 mL with some variability between samples. The larger peak at ~ 45 mL corresponds to the void volume of the column

We initiated the study by testing the ability of ACO to stably bind a series of first row transition metals that serve as cofactors in other oxygenase enzymes, namely, Mn(II), Co(II) and Cu(II). We introduced these metals as well as the native Fe(II) cofactor through their addition to M9 minimal growth media used for the bacterial expression cultures. We omitted metal from one culture to test the ability to produce metal-free apo enzyme. SDS-PAGE analysis of solubilized protein following ammonium sulfate precipitation of the cell lysate supernatant demonstrated that ACO produced with different treatments was expressed at levels comparable to that obtained from Fe(II)-supplemented cultures, with the exception of the sample obtained from manganese-containing culture, whose higher ACO expression level was attributable to the greater cell density obtained in this media (Fig. 1a). Notably, samples produced by minimal media expression had improved initial ACO protein purity following ammonium sulfate fractionation relative to comparable samples obtained from LB media [18]. Further purification by gel-filtration chromatography revealed that all samples exhibited similar elution profiles but with varying amounts of protein eluting in the peak corresponding to non-aggregated ACO (Fig. 1b). These data indicate that ACO expression is not markedly affected by depletion of native Fe(II) or supplementation with non-native metals although final protein yields were lower for ACO expressed in the presence of copper or without added metal.

### Non-native metals are incorporated into ACO during expression

To determine whether metals added to the *E. coli* cultures were incorporated into ACO, we analyzed each purified protein sample by multi-element inductively coupled plasma-optical emission spectroscopy (ICP-OES). We used the experimentally determined ACO molar absorptivity at 280 nm to calculate the sample protein concentrations and ensure an accurate metal:protein stoichiometry [27]. Protein purified from LB media revealed a slight excess of iron in the sample compared to the ACO concentration suggesting either adventitious iron binding to ACO or the presence of iron bound to contaminating proteins (Table 1). None of the other examined metals were present in the sample in significant amounts, except for a small amount of contaminating copper. By contrast, ACO from M9 minimal media supplemented with Fe(II) exhibited ~ 70% iron occupancy. The slightly sub-stoichiometric iron occupancy in this sample is consistent with studies on other Fe(II) dependent dioxygenases expressed in Fe-supplemented minimal media [25]. Likewise, ACO from Co(II)- or Cu(II)-containing cultures displayed levels of target metal incorporation of 0.8 and 0.63, respectively (Table 1), suggesting that these metals could occupy the ACO metal-binding center. Little or no iron was found in either the Co or Cu preparations. By contrast, the Mn(II) preparation displayed only minimal manganese incorporation (1.2%), suggesting that Mn(II) has properties disfavoring its binding to the CCO metal-binding site. Notably, the sample produced in media without added metal contained negligible levels of all metals examined, demonstrating the feasibility of producing metal-free apo-ACO in a soluble form.

### Non-native metals can occupy the ACO active site but do not support catalytic activity

**Fig. 2:**
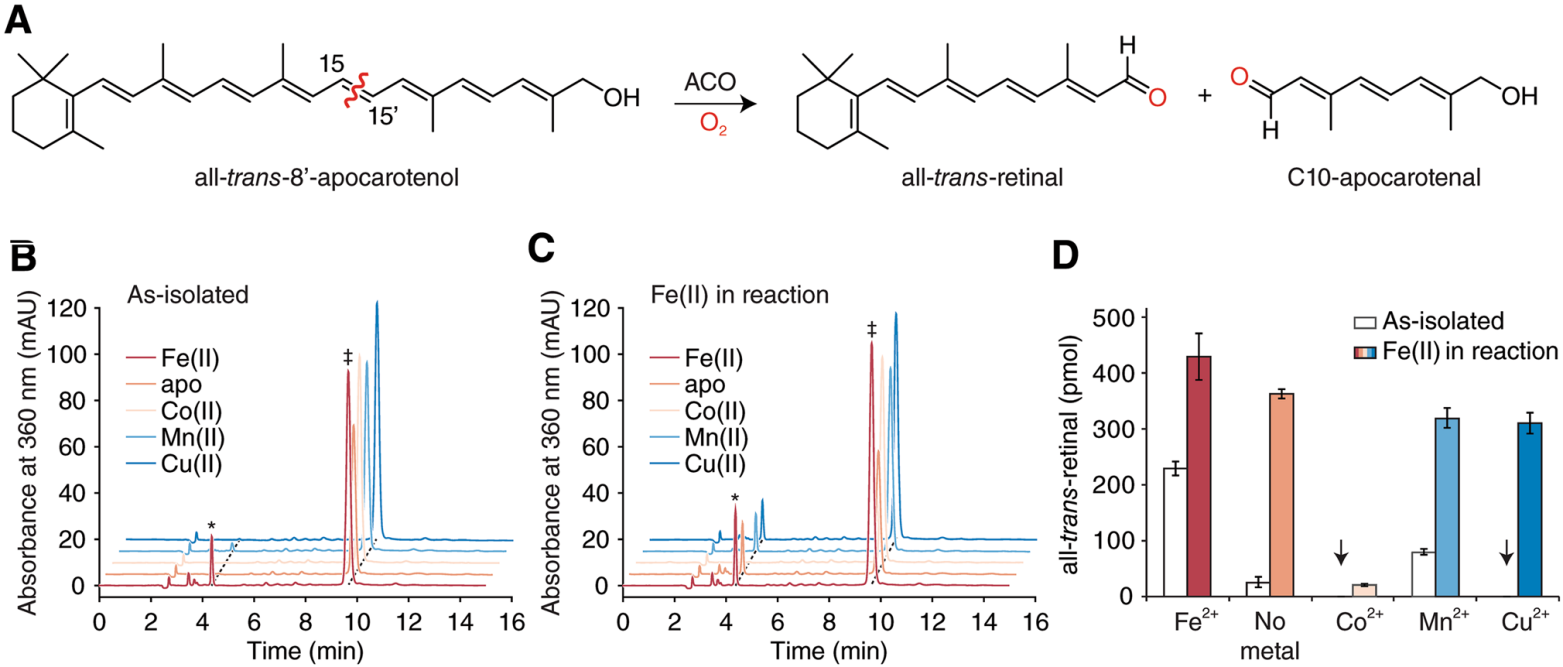
Enzyme activity analysis of apo-, native- and metal-substituted ACO by HPLC. **a** Reaction scheme showing the retinal-forming activity of ACO using the established substrate, all-*trans*-apo-8′-carotenal. **b** and **c** show representative HPLC analyses of the retinal product generated by ACO samples that were produced in the presence or absence of Fe(II) or non-native metals. The reactions were performed without (**b**) and with (**c**) Fe(II) in the assay mixture to ascertain metal binding site accessibility. The peak representing all-*trans*-retinal product is denoted by an asterisk, while the peak corresponding to all-*trans*-apo-8′-carotenal is denoted by a double dagger. The results are quantified in **d**. Data are shown as mean ± SD calculated from measurements carried out in triplicate

**Fig. 3:**
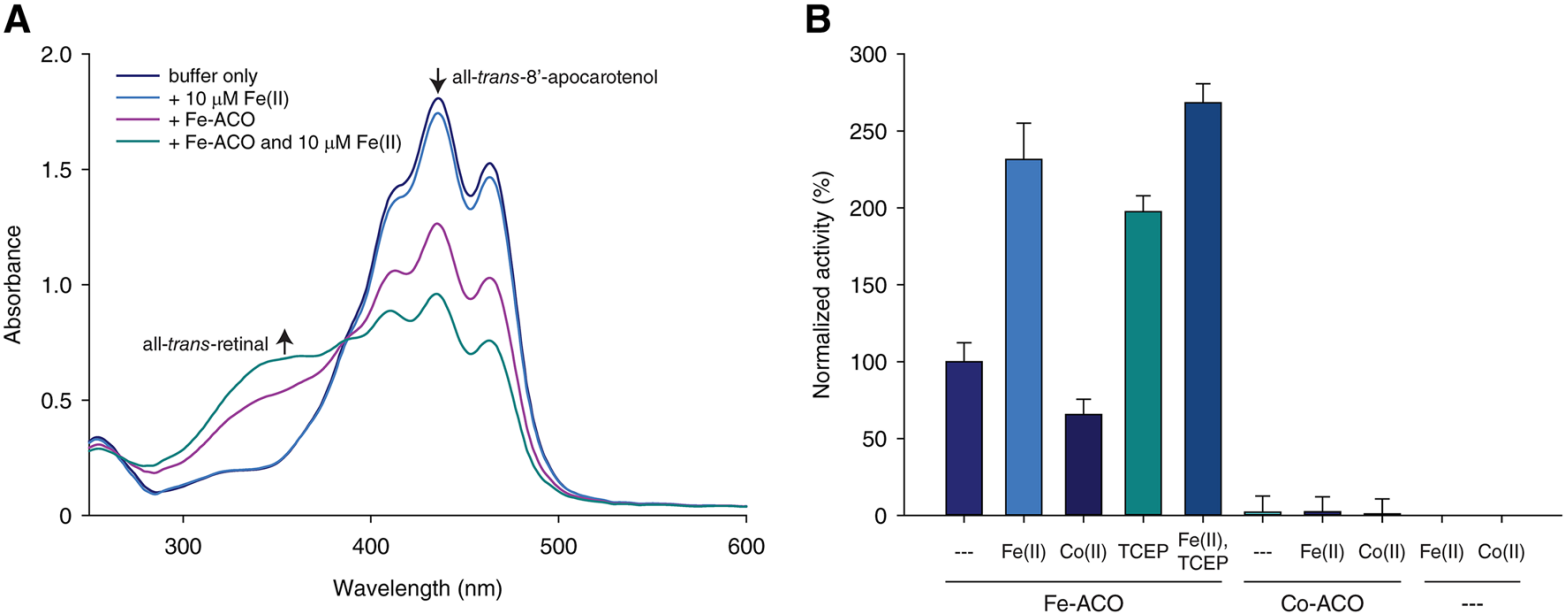
Spectrophotometric assessment of metal and reductant addition on the activity of Fe- and Co-bound forms of ACO. **a** Raw UV/Vis absorbance data recorded in the presence and absence of Fe-ACO with or without added iron. Samples containing Fe-ACO show a reduction in substrate absorbance at 425 nm with a corresponding increase in product absorbance at 350 nm following the 10 min incubation period, whereas iron alone caused negligible loss of substrate absorbance. Samples were pre-incubated for 10 min at room temperature and spectra were recorded following a 10 min reaction period. **b** Relative activity levels of ACO samples pre-incubated with metals or the reductant, Tris-carboxyethyl phosphine (TCEP). Samples with only metals added were pre-incubated at room temperature for 22 h to allow time for potential metal exchange, whereas samples containing TCEP with or without added iron and those containing iron- and TCEP-only were pre-incubated at 28 °C for 10 min prior to initiation of the assay. Assays were performed in triplicate at 28 °C. The results are displayed as mean ± SD

Previously, we showed that the stilbene-cleaving CCO, CAO1, is catalytically inert when prepared in Co(II) substituted form [20]. However, we considered the possibility that metal-substituted ACO may retain catalytic activity due to the significant chemical differences between carotenoids and stilbenoids, as well as electrostatic differences at the metal center observed between CAO1 and ACO by Mössbauer spectroscopy [20]. Moreover, other redox-active metals such as copper may afford a catalytically active enzyme. To test these hypotheses, we measured the ability of the various metal-bound forms of ACO to cleave one of its established substrates, all-*trans*-8′-apocarotenal, leading to the formation of all-*trans*-retinal [8] (Fig. 2a). As expected, ACO expressed in the presence of Fe(II) displayed activity towards this substrate (Figs. 2b, 3a). By contrast, the activity of Mn(II) and metal-free ACO were drastically lower in comparison, the residual activity being attributable to contaminating iron in the preparations (Fig. 2b and Table 1). We also found that Co(II)- and Cu(II)-ACO preparations were virtually devoid of carotenoid cleavage activity, despite the target metals being present in the samples. To test the stability of metal binding, we also carried out assays in the presence of 10 μM Fe(II) (Figs. 2c, 3a). We observed elevated activity for Fe(II)-ACO and metal-free ACO under these conditions consistent with the existence of vacant metal-binding sites in those samples that can be occupied by the added Fe(II). Notably, incubation with Fe(II) alone had a minimal effect on the apocarotenoid absorbance spectrum ruling out non-enzymatic chemistry as the cause of the enhanced retinal formation (Fig. 3a). The ability of apo-ACO activity to be stimulated by externally added ferrous iron to the maximum level of Fe-ACO demonstrated that the apo enzyme is structurally intact with a fully accessible metal-binding site (Fig. 2c). Notably, the activity augmentation for the Fe-ACO sample (~ 2-fold increase) was somewhat greater than that expected from the apparent number of vacant active sites available in the sample (~ 30% of total ACO, Fig. 2d and Table 1). This finding could be explained by the presence of active site Fe(III) in a portion of the ACO sample that can be reduced or displaced by Fe(II) to give an active enzyme. Indeed, we observed that Fe-ACO activity could be enhanced by addition of another reducing agent, Tris-carboxyethyl phosphine (TCEP), as previously described for other CCOs (Fig. 3b) [20, 39]. Interestingly, among the three samples prepared with non-native divalent metals, only the Co-ACO sample remained inactive upon addition of ferrous iron, whereas manganese- and copper-containing samples displayed activity augmentation comparable to apo-ACO (Fig. 2b, c). To further probe the interaction of cobalt with ACO, we tested the effects of Fe(II) and Co(II) addition on the activity of Co-ACO and Fe-ACO to gauge the susceptibility of the metals loaded into the protein during expression to be displaced (Fig. 3b). We found that prolonged incubation of Fe-ACO with 100 μM Co(II) led to a roughly 30% decrease in catalytic activity, whereas incubation of Co-ACO with 100 μM Fe(II) for an equal period of time did not enhance catalytic activity. Taken together with the ICP-OES analyses, these data indicate that iron, cobalt and copper all can occupy the ACO metal binding site and suggest that of the tested non-native metals the enzyme binds cobalt with highest affinity. However, only Fe(II) was able to support ACO catalytic activity.

### Characterization of Co(II)-ACO by optical spectroscopy suggests a five-coordinate metal complex

**Fig. 4:**
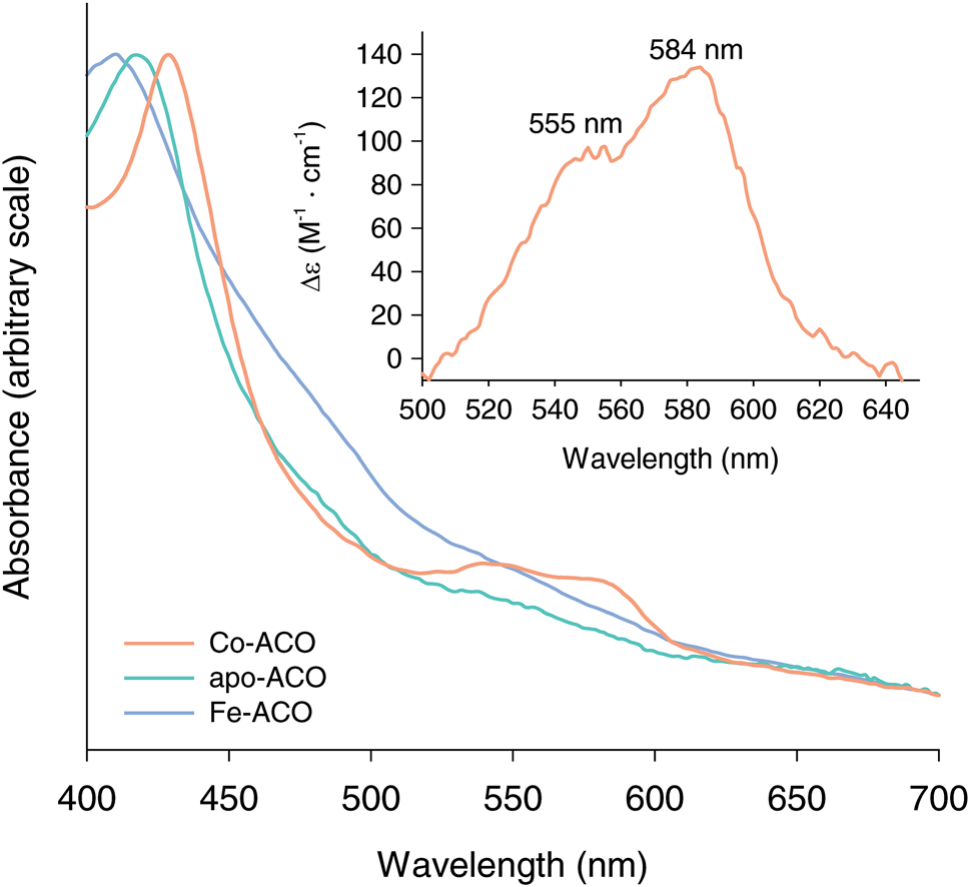
Optical spectroscopy of Co-ACO. Spectra for apo-ACO, Co-ACO, and Fe-ACO are shown as green, orange and slate colored lines, respectively. The spectra have been scaled to their 410–429 nm absorption peaks to facilitate comparison of absorption features in the 500–600 nm region of interest. The inset shows the (Co-ACO–apo-ACO) difference spectrum with the molar absorptivity calculated on the basis of the cobalt concentration in the sample

Whereas apo-ACO was pale yellow, purified Co-ACO exhibited a bronze color that could be partially attributed to light absorption by the bound transition metal. High spin Co(II) *d*–*d* electronic transitions, which occur in the visible region of the electromagnetic spectrum, are spin-allowed but Laporte-forbidden or partially forbidden depending upon whether or not the metal ligand environment is centrosymmetric [40]. Owing to these selection rules, the strength of high spin Co(II) optical absorption bands, which are typically centered in 500–600 nm wavelength range, are correlated with the coordination number and symmetry of the metal ion [23, 41]. We recorded electronic absorption spectra on concentrated samples of Co- and apo-ACO to evaluate the absorptivity of the Co(II) ion in the CCO His-rich ligand environment. Besides the main protein-related peak at ~ 280 nm, Co-ACO exhibited additional features at 429, 540 nm and 575 nm, whereas apo-ACO showed only a 417 nm peak whose height was about three times smaller than that of the Co-ACO 429 nm peak at equal protein concentrations. Fe-ACO also exhibited a similar but broader absorption peak at 410 nm comparable in height to the Co-ACO 429 nm peak at equal protein concentrations (Fig. 4). The identities of the chromophores giving rise to these 410–429 nm absorption peaks remain obscure although the similarity in absorbance maxima suggest a related origin that is modulated by the metal content of the samples. The apo-ACO 417 nm and Co-ACO 429 nm absorption bands possessed tails that extended into the 500–600 nm range adding background to the bands of interest. We removed this background through scaling the apo-ACO spectrum so that its 417 nm absorbance level matched that of the Co-ACO 429 nm absorbance followed by subtraction from the Co-ACO spectrum (Fig. 4). The resulting difference spectrum was bimodal with a minor peak at 555 nm and a major peak at 584 nm, the latter giving a molar absorptivity of 134 M^−1^ cm^−1^ on the basis of the cobalt concentration within the sample (Fig. 4, inset). The spectral shape closely resembled that of octahedral high-spin Co(II) model compounds, which undergo two electronic state transitions elicited by ~ 500–600 nm photons, whereas the extinction coefficient value was in the middle of the range expected for a five coordinate Co(II) center [23, 41]. These findings suggest that one or possibly two additional ligands besides the four protein-associated imidazoles occupy the Co(II) coordination sphere. The absence of an absorption band in the ~ 670–833 nm region of the spectrum, corresponding to a ^4^A′_2_ → ^4^E′ electronic transition, argues against a *D*3_h_-symmetric site, whereas three transitions in the 500–600 nm range are theoretically expected for a high spin Co(II) in a *C*_4v_-symmetric environment [41]. For the latter case, it is conceivable that two of these transitions are close in energy for the CCO Co(II) complex and thus not resolved leading to the observed bimodal absorption feature.

### Crystal structure of Co-ACO reveals a structurally preserved active site compared to Fe-ACO

**Fig. 5:**
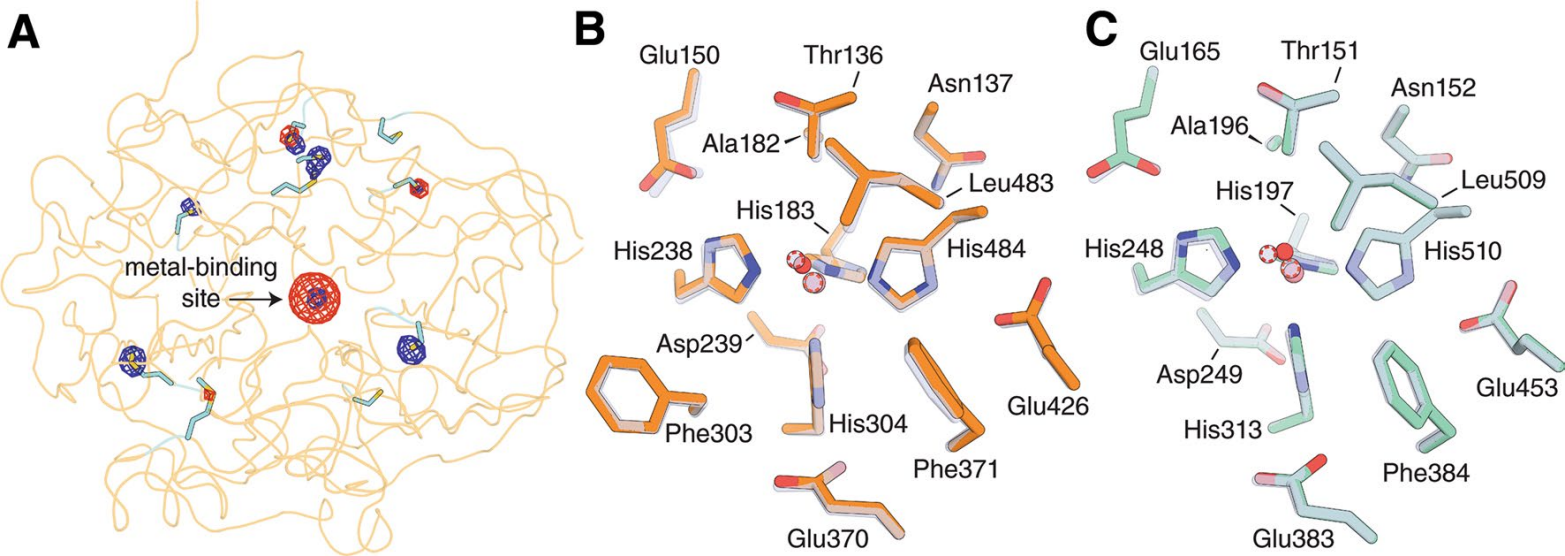
Crystal structures of cobalt-substituted CCOs. **a** Confirmation of active-site bound cobalt in Co-ACO crystals. NCS-averaged anomalous log-likelihood gradient maps were calculated with data collected above (red mesh) and below (blue mesh) the cobalt K absorption edge. Both maps are contoured at 9 RMSD. Anomalous scattering in the vicinity of the sulfur atoms of Met and Cys residues (shown as sticks) demonstrates comparable quality of the pre- and post-edge data sets. The much stronger density peak at the metal binding site in the post-edge map demonstrates the presence of cobalt. **b** Comparison of the active site structure of Co-ACO (orange sticks, salmon and red spheres for Co and solvent, respectively) and Fe-ACO (grey sticks, grey spheres circumscribed with orange and red dashed lines for Fe and solvent, respectively). **c** Comparison of the active site structure of Co-CAO1 (aqua sticks, salmon and red spheres for Co and solvent, respectively) and Fe-CAO1 (grey sticks, grey spheres circumscribed with orange and red dashed lines for Fe and solvent, respectively)

The ICP-OES, catalytic activity and optical spectroscopy data provided strong evidence for the binding of Co(II) at the ACO active center. We confirmed this by crystallizing ACO expressed in the presence of Co(II) and determining its structure through X-ray diffraction (Table 2). Crystallization conditions were identical to those used for native Fe-ACO and no extra Co(II) was supplied in the crystallization cocktail. In this putative Co-substituted ACO structure, a strong *F*_*o*_–*F*_*c*_ electron density peak was present at the metal-binding site indicative of a bound metal within the catalytic center. To verify its identity as cobalt, we collected anomalous diffraction data above and below the cobalt K absorption edge allowing the calculation of cobalt-specific difference electron density maps (Table 2). The metal-binding site contained a strong peak in the post-edge, NCS-averaged, anomalous log-likelihood gradient map, whereas the corresponding peak in the pre-edge map was much weaker (Fig. 5a). The difference in scattering strength could not be attributed to error differences between the two data sets as the anomalous scattering associated with sulfur atoms of Cys and Met was comparable between the two maps (Fig. 5a). These findings supported the modeling of cobalt within the ACO active site. After refinement, the *B*-factor of the cobalt atom was similar to the surrounding ligand atoms in all four molecules of the asymmetric unit, suggesting near complete occupancy of cobalt in the active site of the crystallized protein. This finding contrasts with an ~ 80% cobalt occupancy in the Co(II)-ACO sample as determined by ICP-OES analysis (Table 1), which may indicate preferential incorporation of Co-loaded protein molecules into the crystal.

We next analyzed the structural consequences of cobalt substitution into the ACO active site. The structure of Co-ACO is nearly indistinguishable from that of Fe-ACO based on visual inspection with an overall root-mean-square (RMS) difference between Cα atoms of 0.4 Å, comparable to the RMS differences between monomers within each of the asymmetric units (Fig. S1a and b). The most variable regions included residues 205–211 and 229–234 which comprise the top face of propeller blade II and were previously shown to exhibit flexibility [18], and residues 119–123 which are located in an α-helical segment. Both regions are involved in ACO membrane association and do not contribute to active site structure. With regard to the active site tunnel, only Phe236 showed a consistent conformational difference between the two structures wherein the Cβ–Cγ bond was rotated by ~ 30° resulting in a slightly different orientation of the phenyl ring. Previously, we showed that Phe236Ala mutant ACO is catalytically active, which argues against a subtle rotation of the Phe236 side chain leading to an inactive enzyme [27]. Focusing on the metal center, the coordination geometry and conformations of first and second sphere iron-coordinating residues were indistinguishable between the two metal-bound forms (Fig. 5b). The metal center of Co-ACO is five-coordinate with distorted *C*_4v_ symmetry in agreement with the preceding optical absorption data. We did observe a slight contraction of the His–metal and solvent–metal bond lengths in Co-ACO as compared to Fe-ACO although these differences were not significant within the coordinate uncertainties (Table 3).

**Table 3:** ACO and CAO1 metal–His bond lengths derived from XAS and XRD studies

	Co-ligand bond lengths (Å)		Fe-ligand bond lengths (Å)	
	XRD (6BIG)^a^	XAS^c^	XRD (4OU9)^d^	XAS^d^
ACO				
His183	2.13 ± 0.03^b^	2.10	2.19 ± 0.02	2.15
His238	2.21 ± 0.03		2.25 ± 0.04	
His304	2.17 ± 0.01		2.19 ± 0.04	
His484	2.23 ± 0.03		2.23 ± 0.02	
Solvent	1.96 ± 0.18	1.95	2.08 ± 0.06	2.01

	XRD (6B86)	XAS^c^	XRD (5U8X)^e^	XAS^e^
CAO1				
His197	2.04 ± 0.03	2.09	2.43 ± 0.06	2.15
His248	2.11 ± 0.02		2.17 ± 0.06	
His313	2.03 ± 0.03		2.34 ± 0.03	
His510	2.13 ± 0.01		2.25 ± 0.05	
Solvent	1.96 ± 0.11	1.95	2.45 ± 0.02	1: 1.99 2: 2.51

^a^PDB accession codes are shown in parentheses for X-ray diffraction (XRD) structures. Maximum likelihood coordinate uncertainty estimates are as follows: Co-ACO (6BIG), 0.177 Å; Fe-ACO (4OU9), 0.1 Å; Co-CAO1 (6B86), 0.164 Å; Fe-CAO1 (5U8X), 0.137 Å

^b^Values are the averages and standard deviations computed from the four molecules in the asymmetric unit of each structure

^c^From Tables 4 and 5 of the present work

^d^From Ref. [18]

^e^From Ref. [20]

### Cobalt substitution preserves active site structure in a stilbene-cleaving CCO

We previously showed that Co substitution in the stilbene-cleaving CAO1 results in undetectable catalytic activity [20] as we have observed here for ACO. Although the previously reported crystal structure of Co-CAO1 did not show notable structural differences compared to that of Fe-CAO1, the resolution and data quality of the former structure was substantially inferior compared to the latter [20]. To allow a more rigorous comparison, we collected data on a Co-CAO1 to a higher than previously achieved resolution, with significantly improved quality indicators (Table 2). Similar to ACO, we found only minor structural differences in active site residues between Co-CAO1 and Fe-CAO1 (Fig. 5c and Fig. S1c and d). Notably, in each monomer of the asymmetric unit, cobalt was consistently bound deeper within the 4-His metal binding pocket, in closer proximity to His197 by ~ 0.4 Å relative to iron. Additionally, we observed subtle variability in the positioning of the coordinated solvent between the two metal-bound forms of the enzyme. Like in ACO, we observed a shortening of the metal–His bond lengths in cobalt-substituted CAO1 relative to the native iron form, although the bond lengths for Fe-CAO1 could be artificially elongated owing to submaximal iron occupancy [20]. By contrast, the cobalt ions in Co-CAO1 have *B*-factors that are comparable to the coordinating His N^ε^ atoms indicating full cobalt occupancy at the metal-binding site. Taken together with the preceding structural data on ACO, cobalt substitution appears to preserve active site structure in CCOs without supporting catalytic activity.

### XAS confirms structural similarity of Co-ACO and Co-CAO1 versus Fe counterparts but with slightly contracted metal–ligand bond lengths

**Fig. 6:**
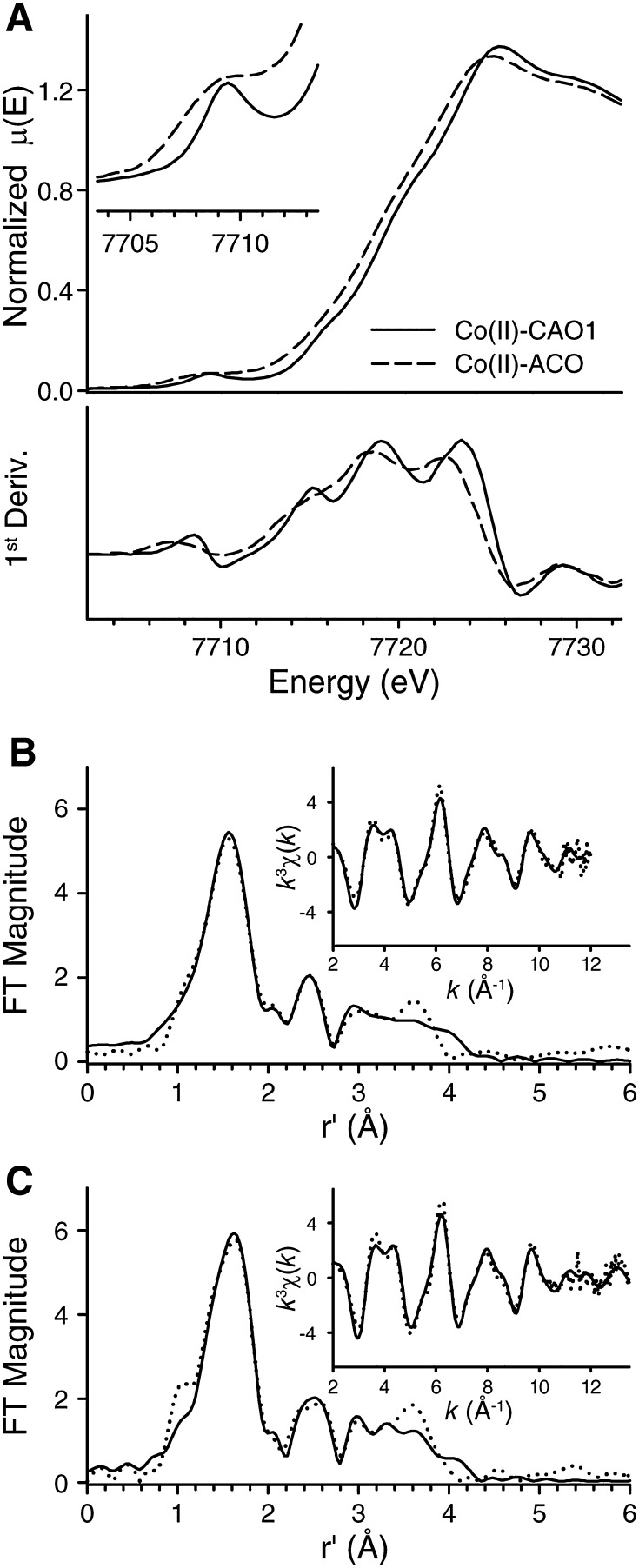
XAS analysis of Co-ACO and Co-CAO1. **a** Normalized XANES spectra of as-isolated Co(II)-CAO1 (solid line) and Co(II)-ACO (dashed line). The inset shows an expansion of the pre-edge region. Representative best fits (bolded entries in Tables 4, 5) to *k*^3^-weighted EXAFS data of Co(II)-ACO (**b**), and Co(II)-CAO1 (**c**). Experimental data are shown as dotted lines, while the best fits are shown as solid lines

The X-ray diffraction data presented in the preceding sections suggested some subtle structural differences between the Fe- and Co-forms of ACO and CAO1, but the limited resolution of the data precluded a definitive assessment. Additionally, the coordination number and symmetry of the cobalt center in Co-ACO was left somewhat ambiguous by the preceding optical absorption data. We addressed both of these remaining issues by recording cobalt K-edge XAS spectra on concentrated Co-ACO and Co-CAO1 samples. Analysis of pre-edge absorption features provides information on the metal site symmetry and coordination number, whereas the extended x-ray absorption fine structure (EXAFS) provides accurate bond lengths for the metal center as it exists in solution. X-ray absorption near-edge spectroscopy (XANES) of both Co-ACO and Co-CAO1 suggested that both enzymes contain Co(II) centers having similar geometric and electronic structures (Fig. 6a). Both Co-substituted proteins exhibit a single 1*s*-to-3*d* pre-edge transition at ca. 7709 eV that can be fit to a single Gaussian-type function with integrated peak areas of 11.6 for Co-CAO1 and ~ 13 for Co-ACO. These pre-edge areas are on the high side of reported Co(II) pre-edge peak areas, which typically range between 3–6 for six-coordinate sites and > 10 for lower symmetries [42–44]. Our pre-edge analysis, therefore, supports a five-coordinate geometry for the cobalt site in both CAO1 and ACO. Of note, the sharper peak shape for Co-CAO1 likely reflects the higher resolution Si(220) monochromator crystal set used for collection of this data set. Both Co-ACO and Co-CAO1 also exhibit two distinct edge inflections (7718.5/7722.5 eV for Co-ACO and 7719.0/7723.5 eV for Co-CAO1), paralleling the spectral shapes seen for the native Fe(II)-containing forms [20, 45]. The blue-shift of the Co-CAO1 edge implied a somewhat higher effective nuclear charge (*Z*_eff_) for this enzyme’s Co(II) center compared to that of ACO, with a similar trend also observed for the native Fe(II) preparations [20]. A more electropositive environment at the metal-binding site for CAO1 compared to ACO was also previously noted from Mössbauer spectroscopy isomer shifts, which reflect s orbital electron density at the nucleus [20]. However, we note that this apparent electronic structure difference is not reflected in the geometric structure seen by X-ray diffraction (XRD) or XAS (vide infra) within the resolution limitations of available data.

EXAFS analysis confirmed the structural model for the cobalt center derived by XRD (Fig. 6b, c and Table 3). The first coordination shells of both Co-ACO and Co-CAO1 can be adequately fit with a disordered single shell of 5–6 N/O scatterers at ca. 2.06 Å (Tables 4, 5). However, in both cases splitting the first coordination shell into a 5-coordinate shell consisting of 4 Co–N/O scatterers at ca. 2.10 Å and 1 Co–O/N scatterer at 1.95 Å elicits a substantial improvement in fit quality and reduction in the magnitude of the Debye–Waller factors. We ascribe the longer scatterers to protein-derived His residues, while the shorter Co–O bond likely corresponds to a solvent-derived hydroxo ligand. Evaluation of outer shell features via multiple scattering methods confirms the presence of four His-derived imidazole units bound to the cobalt center in each enzyme. Our EXAFS results confirm that the crystallographically derived structures are retained in solution, as both Co-ACO and Co-CAO1 show nearly superimposable distorted five-coordinate cobalt sites with a modest ~ 0.05 Å reduction in average M(II)–N_His_ and M(II)–O_solvent_ bond lengths going from Fe(II) to Co(II).

**Table 4:** Selected EXAFS fits for Co(II)-ACO

Fit	Co–N/O			Co–O			Co•••His			Δ*E*_0_	*χ* ^2^	*R*
	*n*	*r*	*σ* ^2^	*n*	*r*	*σ* ^2^	*n*	*r*	*σ* ^2^			
1	4	2.06	5.5							− 0.53	129.31	0.0585
2	5	2.06	7.2							− 1.32	89.97	0.0407
3	6	2.06	8.7							− 2.08	92.66	0.0419
*4*	*4*	*2.08*	*3.4*	*1*	*1.92*	*1.2*				− *2.89*	*77.54*	*0.0191*
5	5	2.07	5.4	1	1.90	3.9				− 3.91	111.61	0.0275
6	4	2.08	4.5	2	1.93	6.8				− 4.55	107.08	0.0263
7	4	2.08	3.5	1	1.92	1.2				− 2.65	158.77	0.1852
8	4	2.10	4.6	1	1.95	2.2	3	3.03 3.16 4.27 4.30	6.5 20.0 11.6 6.6	0.92	36.51	0.0180
***9***	***4***	***2.10***	***4.3***	***1***	***1.95***	***2.0***	***4***	***3.04*** ***3.19*** ***4.21*** ***4.30***	***8.3*** ***16.4*** ***6.5*** ***4.9***	***0.61***	***29.79***	***0.0147***

Fitting range was *k* = 2.0–12.0 Å^−1^ (resolution = 0.16 Å) with back transform ranges of 1–2.2 Å for fits 1–6 and 1–4.0 Å for fits 7–9. *r* is in units of Å; *σ*^2^ is in units of 10^−3^ Å; Δ*E*_0_ is in units of eV; *R* represents the fractional misfit of the data, while *χ*^2^ is the *χ*^2^ fitting metric normalized by the number of independent data points in a given fit

The best first-shell single scattering model is italicized, while the best multiple scattering model, corresponding to the best fit curve in Fig. 6b, is given in bold italics

**Table 5:** Selected EXAFS fits for Co(II)-CAO1

Fit	Co–N/O			Co–O			Co•••His			Δ*E*_0_	*χ* ^2^	*R*
	*n*	*r*	*σ* ^2^	*n*	*r*	*σ* ^2^	*n*	*r*	*σ* ^2^			
1	4	2.07	5.4							3.58	84.52	0.0750
2	5	2.07	6.9							2.74	54.93	0.0487
3	6	2.06	8.3							1.98	49.53	0.0440
4	5	2.07	5.2	1	1.91	3.3				0.36	39.27	0.0216
*5*	*4*	*2.08*	*3.4*	*1*	*1.93*	*1.5*				*1.27*	*33.37*	*0.0184*
6	4	2.08	3.7	1	1.93	2.0				1.11	82.60	0.1985
7	4	2.10	3.7	1	1.95	1.8	3	3.05 3.19 4.27 4.37	5.9 19.4 12.2 9.0	3.64	20.94	0.0277
***8***	***4***	***2.09***	***3.8***	***1***	***1.95***	***1.9***	***4***	***3.06*** ***3.22*** ***4.24*** ***4.32***	***7.6*** ***15.7*** 4.8* 4.8*	***3.63***	***17.00***	***0.0248***

Fitting range was *k* = 2.0–13.5 Å^−1^ (resolution = 0.14 Å) with back transform ranges of 1–2.15 Å for fits 1–5 and 1–4.15 Å for fits 6–8. Parameters marked with * were refined to a common value in the fit. *r* is in units of Å; *σ*^2^ is in units of 10^−3^ Å; Δ*E*_0_ is in units of eV; *R* represents the fractional misfit of the data, while *χ*^2^ is the *χ*^2^ fitting metric normalized by the number of independent data points in a given fit

The best first-shell single scattering model is italicized, while the best multiple scattering model, corresponding to the best fit curve in Fig. 6c, is given in bold italics

## Discussion

The preceding results demonstrate the feasibility of substituting non-native metals in carotenoid-cleaving members of the CCO family as shown here for a prototypical member, *Synechocystis* ACO. We also describe the production of essentially metal-free ACO in a folded, soluble form through expression of the protein in un-supplemented M9 minimal media. This work builds upon our prior study on the stilbene-cleaving CCO, CAO1, where it was shown that cobalt could be stably incorporated into the metal-binding site of the enzyme [20]. In the present study we find that cobalt is also effectively taken up into the ACO active site and coordinates tightly to the 4-His metal-binding motif within the active site. Copper also appears capable of occupying the ACO metal-binding site, although we found it could be displaced by iron in contrast to cobalt. Notably, this behavior contrasts with a more stable binding for Cu(II) compared to Co(II) expected from the Irving–Williams series [46]. Interestingly, we were unable to achieve effective manganese substitution even with high levels of this metal added to the bacterial culture. We cannot attribute this to reduced cell viability as manganese supplementation was found to boost growth rate and final cell density. Manganese binding is favored at six-coordinate, oxygen/His sites [47], which are provided by facial triad enzymes [25, 48, 49]. By contrast, the canonical 4-His metal-binding motif of CCOs admits only a single solvent-derived oxygen into the coordination sphere, which could disfavor manganese incorporation.

Metal substitution strategies have been employed in a number of other mononuclear non-heme iron proteins as a means to investigate their catalytic and structural properties. Cobalt-substitution in particular has been successfully employed for proteins belonging to well-characterized non-heme iron enzyme families. In the case of the Rieske enzyme, dicamba dioxygenase [21], as well as TauD [50], a member of the α-ketoglutarate/taurine-dependent dioxygenase family, cobalt substitution results in catalytically inert enzymes. In both cases, the activation of dioxygen involves formation of high-valent iron-oxy species [51]. The inability of Co(II) to functionally substitute in these enzymes can thus be rationalized from the inaccessibility of cobalt to these higher oxidation states due to its elevated reduction potential relative to iron. Likewise, Co(II) substitution in the intradiol-cleaving protocatechuate-3,4-dioxygenase results in an inactive enzyme [52] owing to the requirement of trivalent iron for the initial step of catalysis and the reduced ability for cobalt to cycle between the +2 and +3 oxidation states under normal biological conditions.

By contrast, the cobalt-substituted forms of 2,3-HPCD [23], a vicinal oxygen chelate enzyme and the cupin-fold ring-cleaving oxygenase, quercetin 2,3-dioxygenase [24, 53], both exhibit robust catalytic activity in relation to their Fe-bound forms. Despite their phylogenic diversity, these enzymes share a common catalytic theme: aromatic alkene bond cleavage of a redox-active substrate via direct substrate coordination of the metal center through one or two phenolic oxygen atoms. Both enzymes are thought to use their metal cofactor to channel electrons from the organic substrate to bound dioxygen resulting in simultaneous activation of both substrates with only a transient change in metal redox state [22, 25, 54], which explains the ability of cobalt to support catalysis in these enzymes.

CCOs have features common to both Co-active and Co-inactive dioxygenase enzymes. On one hand, like some Rieske dioxygenases including dicamba dioxygenase, CCOs substrates do not directly coordinate to the metal center when they are bound in the active site. But like the catechol and quercetin dioxygenases, CCO substrates are redox active and possibly do not require a powerful oxidizing agent (e.g., high valent iron-oxy species) for cleavage of the target alkene bond. Thus, it is interesting that Co-substituted forms of both carotenoid- and stilbenoid-cleaving CCOs are catalytically inactive [20]. While our current understanding of the exact iron-oxy species involved in CCO catalysis is limited, these results suggest that the inactivity of Co-CCOs may relate to the necessity for a relatively long-lived iron(III)-superoxo or conceivably a higher valent iron in the CCO catalytic cycle. This difference between CCOs and the Co-active dioxygenases mentioned above could relate to the absence of direct substrate binding to the iron center, which excludes a direct pathway for electron flow, as well as eliminates possible redox tuning of the metal by organic substrate coordination.

The absence of catalytic activity in Co-CCOs could also result from a perturbation in the active site structure upon replacement of the native Fe(II) cofactor. The crystallographic and XAS data that we present have allowed us to rigorously evaluate this potential explanation. Little or no consistent differences in active site residues were observed between the Co- and Fe-bound forms of both ACO and CAO1, indicating that changes in their substrate binding sites due to the presence of non-native metal are not responsible for the absence of activity. We did observe by both X-ray crystallography and XAS a slight but consistent shortening in metal–ligand bond lengths (~ 0.05 Å) in the Co-bound forms of both enzymes relative to their Fe-bound counterparts (Table 2). The shorter metal–ligand bond lengths in Co-CCOs could be at least partially rationalized by the smaller ionic radius of high-spin Co(II) relative to high-spin Fe(II) [55]. Regardless, it seems unlikely that this small difference in metal–ligand bond length on its own could lead to such a profound change in catalytic activity. However, the difference in bond length may reflect varying strength of metal–ligand interactions. Such a difference is suggested by our activity assay data where addition of Fe(II) to Co-ACO had a minimal ability to boost catalytic activity, whereas incubation of Fe-ACO with Co(II) resulted in a substantial suppression in catalytic activity indicative of active site Fe(II) substitution by Co(II) (Fig. 3b).

Co-substituted CCOs may be valuable tools for elucidating biochemical properties and biological roles of these enzymes. We previously showed that Co-substitution in CAO1 allows for stable trapping of active site-bound substrate for structural characterization. Co-substitution in carotenoid-cleaving CCOs, as shown to be feasible in the present study, is likely to facilitate three-dimensional structure determination of CCO–carotenoid Michaelis complexes, which to date have evaded characterization. This goal will, however, likely require other advances to overcome the problem of carotenoid aqueous insolubility. As mentioned in the Introduction, the biological substrates of some CCOs remain to be elucidated [8, 10]. Co-substituted CCOs, being catalytically inert but structurally intact, may be helpful for identification of high affinity substrates through affinity chromatography coupled with mass spectrometry. Additionally, Co-substituted CCOs may also be useful for carrying out binding assays in the absence of confounding catalysis to assess differences in substrate affinity.

## Electronic supplementary material

Below is the link to the electronic supplementary material.

Supplementary material 1 (PDF 6353 kb)

## Abbreviations

ACO: *Synechocystis* sp. PCC6803 apocarotenoid oxygenase
BCO: β-Carotene oxygenase
CAO1: *Neurospora crassa* carotenoid oxygenase 1
CCO: Carotenoid cleavage oxygenase
EXAFS: Extended X-ray absorption fine structure
HPLC: High-performance liquid chromatography
ICP-OES: Inductively coupled plasma-optical emission spectroscopy
LB: Lysogeny broth
NSLS: National Synchrotron Light Source
RMS: Root-mean-square
SDS-PAGE: Sodium dodecyl sulfate-polyacrylamide gel electrophoresis
SSRL: Stanford Synchrotron Radiation Lightsource
TCEP: Tris-carboxyethyl phosphine
XANES: X-ray absorption near edge spectroscopy
XAS: X-ray absorption spectroscopy
XRD: X-ray diffraction
