# Supplementary material for: Preparation and characterization of metal-substituted carotenoid cleavage oxygenases
Source: J Biol Inorg Chem. 2018 Jun 26;23(6):887–901. doi: 10.1007/s00775-018-1586-0 (PMC6060882; doi:10.1007/s00775-018-1586-0)
Supplement: Supplementary file 1 — Supplementary material 1 (PDF 6353 kb) [file 775_2018_1586_MOESM1_ESM.pdf]

Supplemental Figure

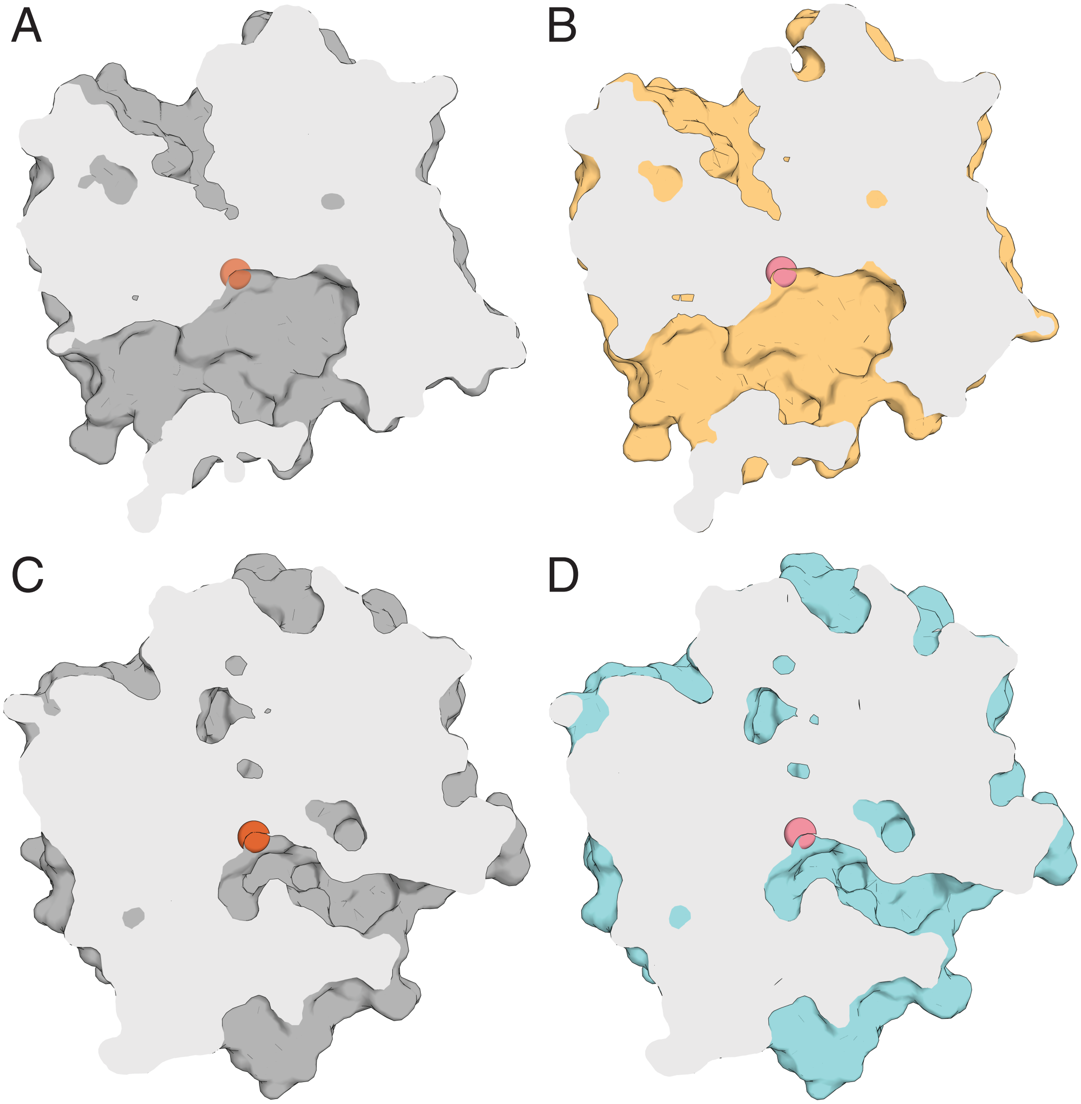

Figure S1 *Cut-away surface views of (**a**) Fe-ACO (PDB accession code 4OU9), (**b**) Co-ACO (PDB accession code 6BIG), (**c**) Fe-CAO1 (PDB accession code 5U8X), and (**d**) Co-CAO1 (PDB accession code 6B86).* Note the close structural similarity in the active site tunnels/cavities in (**a**) versus (**b**) and (**c**) versus (**d**) leading to the bound metals (shown as brown and salmon spheres for iron and cobalt, respectively).
